# Rationally Designed Bi_2_M_2_O_9_ (M = Mo/W) Photocatalysts with Significantly Enhanced Photocatalytic Activity

**DOI:** 10.3390/molecules26237334

**Published:** 2021-12-02

**Authors:** Fangzhi Wang, Xiaoyan Zhou, Jing Li, Qiuyue He, Ling Zheng, Qing Liu, Yan Chen, Guizhai Zhang, Xintong Liu, Hongda Li

**Affiliations:** 1 School of Resources and Environmental Engineering, Shandong Agriculture and Engineering University, Jinan 250100, China; wfz0814@126.com (F.W.); yaling110@163.com (X.Z.); ripplelj@126.com (J.L.); ajheqiuyue@163.com (Q.H.); zhengl1988@126.com (L.Z.); liuqingliuqing2021@163.com (Q.L.); ychen0612@163.com (Y.C.); zgzok2005@163.com (G.Z.); 2 School of Light Industry, Beijing Technology and Business University, Beijing 100048, China; 3 School of Microelectronics and Materials Engineering, Guangxi University of Science and Technology, Liuzhou 545006, China

**Keywords:** Bi_2_W_2_O_9_, Bi_2_Mo_2_O_9_, modified structure, visible light, photocatalysis

## Abstract

Novel Bi_2_W_2_O_9_ and Bi_2_Mo_2_O_9_ with irregular polyhedron structure were successfully synthesized by a hydrothermal method. Compared to ordinary Bi_2_WO_6_ and Bi_2_MoO_6_, the modified structure of Bi_2_W_2_O_9_ and Bi_2_Mo_2_O_9_ were observed, which led to an enhancement of photocatalytic performance. To investigate the possible mechanism of enhancing photocatalytic efficiency, the crystal structure, morphology, elemental composition, and optical properties of Bi_2_WO_6_, Bi_2_MO_6_, Bi_2_W_2_O_9_, and Bi_2_Mo_2_O_9_ were examined. UV-Vis diffuse reflectance spectroscopy revealed the visible-light absorption ability of Bi_2_WO_6_, Bi_2_MO_6_, Bi_2_W_2_O_9_, and Bi_2_Mo_2_O_9_. Photoluminescence (PL) and photocurrent indicated that Bi_2_W_2_O_9_ and Bi_2_Mo_2_O_9_ pose an enhanced ability of photogenerated electron–hole pairs separation. Radical trapping experiments revealed that photogenerated holes and superoxide radicals were the main active species. It can be conjectured that the promoted photocatalytic performance related to the modified structure, and a possible mechanism was discussed in detail.

## 1. Introduction

Semiconductor photocatalysis technology has received increasing attention as a green approach since it can be widely applied in the areas of carbon dioxide reduction, water splitting, and organic pollutants degradation [1,2,3,4]. It is no doubt that TiO_2_ is a popular photocatalyst. However, the band gap of TiO_2_ is so wide that it can only respond to ultraviolet (UV) light. Therefore, tremendous efforts have been made to develop new visible-light-driven (VLD) photocatalysts, such as Bi_2_WO_6_, Bi_2_MoO_6_, MoS_2_, g-C_3_N_4_, BiOBr, BiVO_4_, and so on [5,6,7,8].

Both Bi_2_WO_6_ and Bi_2_MoO_6_ are active members of the Aurivillius oxide family with a special layer structure [9,10,11,12]. Bi_2_WO_6_ and Bi_2_MoO_6_ (Bi_2_MO_6_, M = W/Mo) possess similar crystal structure. Bi_2_WO_6_ consists of WO_6_ layers and [Bi_2_O_2_]^2+^ layers, and Bi_2_MoO_6_ consists of [MoO_2_]^2+^ layers and [Bi_2_O_2_]^2+^ layers. Such a layered structure is favorable to the separation and transfer of photogenerated carriers [13]. Moreover, Bi_2_MO_6_ can absorb visible light and has good stability against photocorrosion. Thus, they have displayed potential photocatalytic performance for the decontamination of contaminants [14,15]. However, their practical application remains limited because of the high recombination rate of photogenerated electron–hole pairs in photocatalytic processes, and the visible-light use efficiency is still limited, which only responds to the light under 500 nm [16,17]. To solve these issues, composite materials with a heterojunction structure, doping of other ions, and loading of noble metal co-catalysts have been extensively investigated [18,19,20,21,22]. The results indicate that these methods essentially changed the structure of Bi_2_MO_6_ crystal to effectively inhibit the recombination of photogenerated electron–hole pairs under charge transmission, resulting in high photocatalytic performance. In this case, finding a kind of bismuthate with appropriate crystal structure is a potential approach to promote photocatalytic performance. Based on the special layer structure of Bi_2_MO_6_, a train of thought to modify the layer structure can be attempted.

The designation of Bi_2_M_2_O_9_ (M = W/Mo) was adopted because of the similar crystal structure between Bi_2_W_2_O_9_ and Bi_2_Mo_2_O_9_. There are similarities and differences between Bi_2_M_2_O_9_ and Bi_2_MO_6_. Both Bi_2_M_2_O_9_ and Bi_2_MO_6_ consist of (Bi_2_O_2_)^2+^ and (M_x_O_y_)^2−^ (M = W/Mo) layers, while the difference is that the (M_x_O_y_)^2−^ (M = W/Mo) layer is (M_2_O_7_)^2−^ in Bi_2_M_2_O_9_ and (MO_4_)^2−^ in Bi_2_MO_6_. Given this kind of difference, a sort of structure modification phenomenon can take place in Bi_2_M_2_O_9_ crystal, and certainly lead to chemical bond changes.

In this study, novel morphology Bi_2_M_2_O_9_ photocatalysts and ordinary Bi_2_MO_6_ were synthesized by a hydrothermal process. The modified structure of Bi_2_M_2_O_9_ can facilitate charge separation to promote photocatalytic performance. Moreover, the modification of structure and chemical bond changes were studied, and the relationship between modified structure and promoted photocatalytic performance was investigated. We hope to explore a potential strategy to obtain a highly efficient visible-light-driven photocatalyst.

## 2. Result and Discussion

### 2.1. XRD Analysis

**Figure 1:**
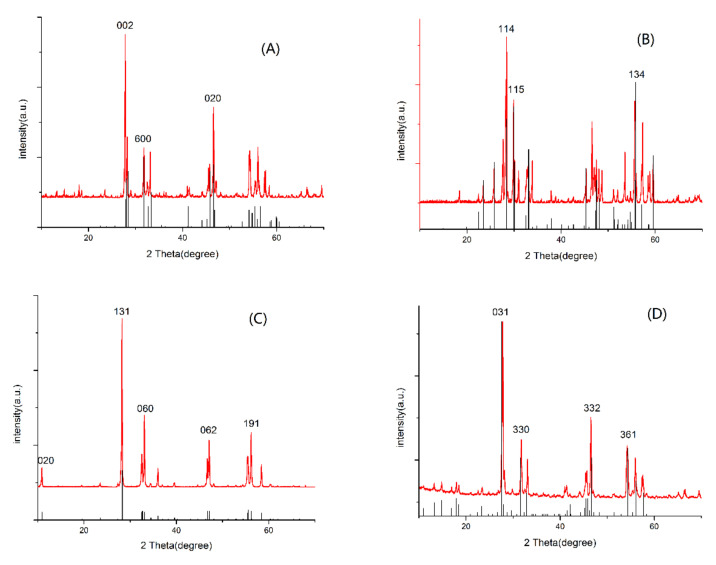
X-ray diffraction patterns of prepared samples (**A**) Bi_2_WO_6__,_ (**B**) Bi_2_W_2_O_9_, (**C**) Bi_2_MoO_6_, and (**D**) Bi_2_Mo_2_O_9_.

The typical diffraction patterns of the as-prepared samples can be observed in Figure 1, which indicates the successful synthesis of samples using the hydrothermal method. It also reveals the crystal style and major diffraction peaks of Bi_2_WO_6_, Bi_2_MO_6_, Bi_2_W_2_O_9_, and Bi_2_Mo_2_O_9_ in panel A to D, respectively. The major diffraction peaks at 2θ values of 27.5°, 33.4°, and 47.1° were indexed to (0 0 2), (6 0 0) and (0 2 0) of Bi_2_WO_6_ in Figure 1A, 2θ values of 27.7°, 29.9°, and 55.7° were indexed to (1 1 4), (1 1 5), and (1 3 4) of Bi_2_W_2_O_9_ in Figure 1B, 2θ values of 10.9°, 28.3°, 33.1°, 47.2°, and 56.2° were indexed to (0 2 0), (1 3 1), (0 6 0), (0 6 2), and (1 9 1) of Bi_2_MoO_6_ in Figure 1C, and 2θ values of 25.8°, 31.8°, 36.9°, and 54.3° were indexed to (031), (330), (332) and (361) of Bi_2_Mo_2_O_9_ in Figure 1D, respectively [23,24,25]. No signal for any crystalline phase of bismuth oxides were observed in the as-prepared photocatalysts.

**Figure 2:**
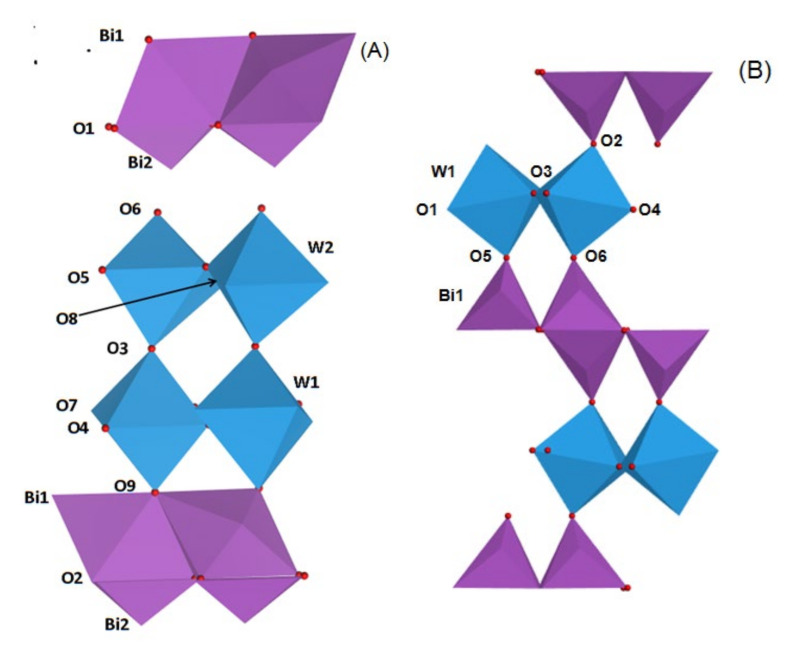
Models of Bi_2_W_2_O_9_ (**A**) and Bi_2_WO_6_ (**B**) consist of (Bi_2_O_2_)^2^^+^ and (W_2_O_7_)^2^^−^ or (WO_4_)^2^^−^ layers.

It was reported that the crystal structure of Bi_2_MO_6_ and Bi_2_M_2_O_9_ photocatalysts can be described as (Bi_2_O_2_)^2+^ layer and (MO_4_)^2−^ or (M_2_O_7_)^2−^ layer alternately connect to each other [26,27]. The polyhedron style model of Bi_2_WO_6_ and Bi_2_W_2_O_9_ were exhibited in Figure 2. As shown in Figure 2A, there is a double W-O layer between two (Bi_2_O_2_)^2^^+^ layers. The W atom and six surrounding oxygen atoms formed a WO_6_ octahedra, which connects with other similar octahedra by axial O3 in a lengthways direction and equatorial O4, O5, O7 and O8 oxygen atoms in a crosswise direction, forming double (W_2_O_7_)^2^^−^ layers. Top and bottom oxygen atoms O6 and O9 are located close to (Bi_2_O_2_)^2^^+^ layers above and below WO_6_ octahedra respectively, which eventually formed Bi_2_W_2_O_9_ consisting of (W_2_O_7_)^2^^−^ and (Bi_2_O_2_)^2^^+^ layers. In addition, as-prepared Bi_2_W_2_O_9_ structure can be regarded as a modification of the tetragonal structure. The formation principle of Bi_2_WO_6_ (Figure 2B) is similar to that of Bi_2_W_2_O_9_. The WO_6_ octahedra join another octahedra by axial O3 in a lengthways direction forming a single (WO_4_)^2^^−^ layer, and equatorial O5, O6, and O2 no longer join other WO_6_ octahedra but join (Bi_2_O_2_)^2^^+^ instead. Bi_2_WO_6_ with a single (WO_4_)^2^^−^ layer is formed in this way.

As a result of the layer structure of Bi_2_WO_6_ and Bi_2_W_2_O_9_, some translational motions of layered crystal can be regarded as a Rigid Unit (RU) layer motion. There are two shared RU modes in Bi_2_W_2_O_9_, and the adjacent layers move parallel to the layer planes, which leads to modified compressional RU modes between adjacent layers in the perpendicular direction of the layer planes. As for Bi_2_WO_6_ structure, there is only one RU mode and the effect of compression between adjacent layers is weaker than that of Bi_2_W_2_O_9_. As a result, compression of Bi_2_W_2_O_9_ layer structure changes the chemical bond properties.

### 2.2. Morphology Characterization

**Figure 3:**
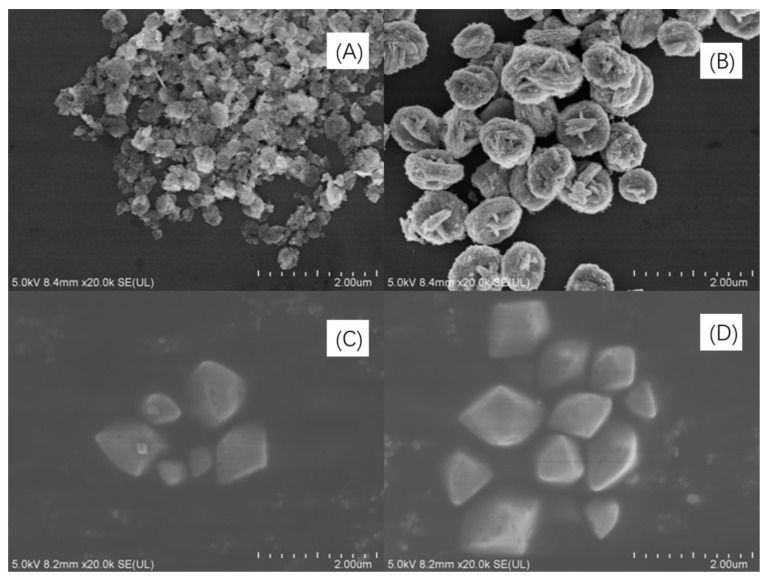
SEM images of (**A**) Bi_2_WO_6_, (**B**) Bi_2_W_2_O_9_, (**C**) Bi_2_MoO_6_ and (**D**) Bi_2_Mo_2_O_9_.

SEM images of Bi_2_WO_6_, Bi_2_W_2_O_9_, Bi_2_MoO_6_, and Bi_2_Mo_2_O_9_ are illustrated in Figure 3 panels a to d, respectively. The morphology of Bi_2_WO_6_ in Figure 3A exhibits fastener-like nanoparticles with the radius of no more than 100 nm. The dimerization bismuthate Bi_2_W_2_O_9_ certainly kept a similar fastener-like morphology as that of Bi_2_WO_6_ (Figure 3B). However, it can be observed that a volume increase phenomenon exists in Bi_2_W_2_O_9_ crystal, and the average radius rose to about 400 nm at the same plotting scale compared to Bi_2_WO_6_. In terms of Bi_2_MoO_6_ and Bi_2_Mo_2_O_9_, Figure 3C,D represent the panoramic SEM images of Bi_2_MoO_6_ and Bi_2_Mo_2_O_9_. Both Bi_2_MoO_6_ and Bi_2_Mo_2_O_9_ are of irregular polyhedron morphology and the variation is that there is also an increase of radius in Bi_2_Mo_2_O_9_ compared to Bi_2_MoO_6_, but the increasing rate is less than that of Bi_2_W_2_O_9_ and Bi_2_WO_6_. Double RU of Bi_2_M_2_O_9_ increase of the distance of adjacent (Bi_2_O_2_)^2^^+^ layers may be the possible reason for the volume augment. The novel morphology observed in SEM images may be attributed to the introduction of AOT surfactant during the synthesis process.

**Figure 4:**
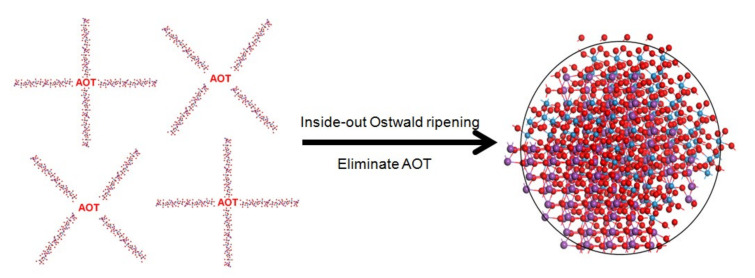
Possible formation process of novel morphology samples with AOT surfactant.

The possible synthesis mechanism is as follows. Raw materials have aggregated together to form some spheres during the hydrothermal process and finally the products have grown into clear-cut fastener-like or irregular polyhedron microspheres. At the beginning of the reaction, Bi^3+^ with (M_x_O_y_) ^2−^ ions precipitated quickly under the driving force of low-solubility products of Bi_2_M_x_O_y_. Subsequently, AOT molecules are absorbed on the surface of nanoparticles through intermolecular interaction, and the newly formed nanoparticles are aggregated into loose microspheres, forming the Bi_2_M_x_O_y_-AOT composite systems. These Bi_2_M_x_O_y_-AOT composite systems connected with each other in various shapes. Finally, these amorphous nanoparticles underwent Ostwald ripening from the inside out as their surfaces come into contact with the surrounding solution. As a result, the internal nanoparticles tend to dissolve, which provides the driving force for spontaneous inside-out Ostwald ripening. This dissolution process could initiate at regions either near the surface or around the center of the microspheres. Redundant AOT was washed by the solution. Ostwald ripening occurred during the synthesis process of Bi_2_WO_6_, Bi_2_W_2_O_9_, Bi_2_MoO_6_, and Bi_2_Mo_2_O_9_ presumably depending on the packing of primary nanoparticles and ripening characteristics of AOT. A simple schematic illustration for the formation of the process is given in Figure 4.

To confirm the atomic composition of Bi_2_WO_6_, Bi_2_W_2_O_9_, Bi_2_MoO_6_, and Bi_2_Mo_2_O_9_ samples conform to the theoretical value, EDX measurement was carried out and the data are listed in Table 1.

The EDX data show that although there is deviation compared to the experimental data with theoretical value, the deviation is within acceptable limits.

### 2.3. Chemical State Analysis

X-ray photoelectron spectroscopy (XPS) analysis is used to further investigate the chemical state and surface chemical composition of Bi_2_WO_6,_ Bi_2_W_2_O_9_, Bi_2_MoO_6_, and Bi_2_Mo_2_O_9_, especially to understand the structure modification effect of Bi_2_W_2_O_9_ and Bi_2_Mo_2_O_9_ on the binding energy, which has a great influence on photocatalytic performance [28].

**Figure 5:**
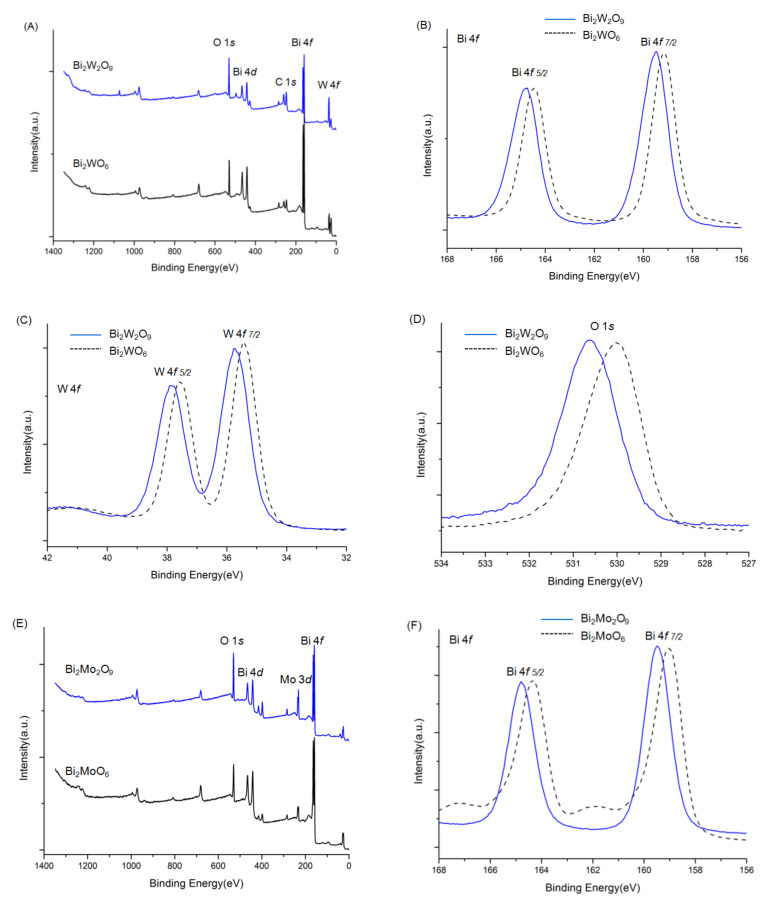

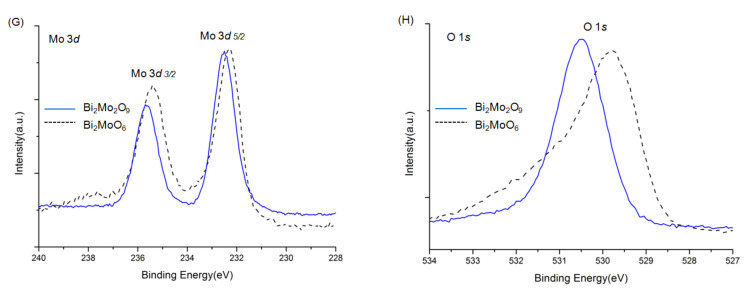
XPS spectra of Bi_2_WO_6,_ Bi_2_W_2_O_9_, Bi_2_MoO_6_ and Bi_2_Mo_2_O_9_: ((**A**): Bi_2_WO_6_ and Bi_2_W_2_O_9_) ((**E**): Bi_2_W_2_O_9_) overall spectra; (**B**,**F**) Bi 4*f*; (**C**) W *f*; (**D**,**H**) O 1*s*; (**G**) Mo 3*d*.

The overall XPS spectra of Bi_2_WO_6,_ Bi_2_W_2_O_9_, Bi_2_MoO_6_, and Bi_2_Mo_2_O_9_ are shown in Figure 5. The characteristic peaks of the Bi, W, Mo, and O elements were detected. Before the analysis, all peaks of the other elements were calibrated according to the deviation between the C 1*s* peak and the standard signal of C 1*s* at 284.8 eV [29]. No XPS characteristic peaks of N 1*s* were detected at around 400 eV, although raw material contained nitrogen, which indicated no nitrogen was doped in Bi_2_WO_6,_ Bi_2_W_2_O_9_, Bi_2_MoO_6_, and Bi_2_Mo_2_O_9_ samples. The signals of Bi_2_WO_6_ in Figure 5B were attributed to Bi 4*f*_7/2_ and Bi 4*f*_5/2_ states respectively at 159.2 and 164.5 eV [30]. The binding energy of W 4*f*_7/2_ and W 4*f*_5/2_ were observed at 35.4 and 37.6 eV (Figure 5C) that can be attributed to W^6+^ [31]. The binding energy peak in Figure 5D located at 530.0 eV corresponds to O 1*s* state in Bi_2_WO_6_ [32]. The same examined element spectra in Bi_2_W_2_O_9_ were exhibited together with those in Bi_2_WO_6_ in the same panels. It can be obviously observed that all Bi, W, and O have similar peak patterns, and the difference is that characteristic peaks of Bi_2_W_2_O_9_ shift towards higher binding energy, which illustrates that the chemical environment has changed, and a higher binding energy indicates the existence of the electron-drawing group. The same phenomenon can be observed in Bi_2_MoO_6_ and Bi_2_Mo_2_O_9_ in panels E to H, where Bi, Mo, and O elements in Bi_2_Mo_2_O_9_ pose a higher binding energy.

### 2.4. Optical Properties

The optical properties of Bi_2_WO_6,_ Bi_2_W_2_O_9_, Bi_2_MoO_6_, and Bi_2_Mo_2_O_9_ were characterized through a UV-Vis diffuse reflectance spectrometer in the wavelength range of 250–800 nm [33]. Compared to Bi_2_WO_6_ and Bi_2_MoO_6_, it can be observed obviously that there is a red shift of the light absorption edge from Bi_2_W_2_O_9_ and Bi_2_Mo_2_O_9_ samples, respectively. The optical band gap of the as-prepared photocatalysts was calculated using the following Equation (1) [34].

**Figure 6:**
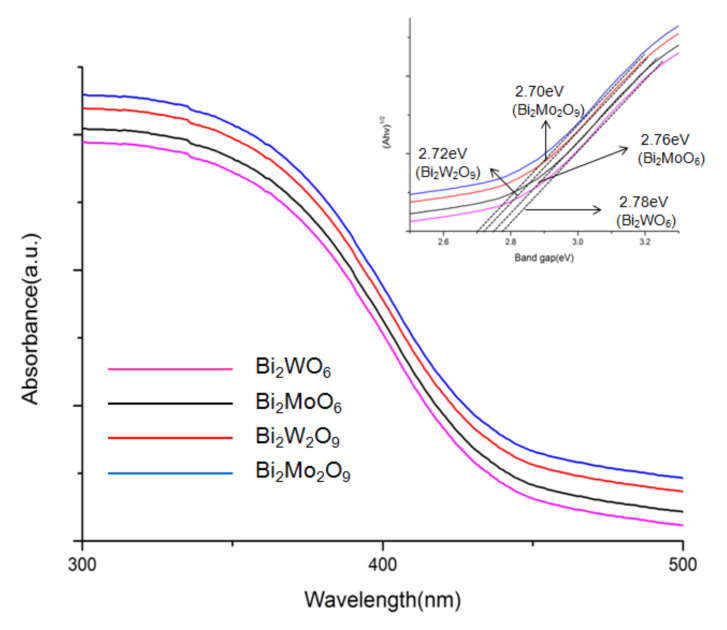
DRS spectra of Bi_2_WO_6,_ Bi_2_W_2_O_9_, Bi_2_MoO_6_ and Bi_2_Mo_2_O_9_. The inset shows the band gap energies.

Ahν = α(hν − E_g_)^n/2^ (1)

in which h, α, A, ν, and E_g_ represent Planck’s constant with the unit of eV, a constant, the absorption coefficient near the absorption edge, light frequency, the absorption band gap energy, respectively, and n is equal to 1 or 4, depending on whether the optical transition type is direct or indirect. Bi_2_WO_6,_ Bi_2_W_2_O_9_, Bi_2_MoO_6_, and Bi_2_Mo_2_O_9_ have a direct band gap, and n is 1 herein. The inset shows the curve of (αhν)^2^ versus hν for the as-prepared samples. It can be observed that an evidential red shift exists in Bi_2_M_2_O_9_ samples compared to Bi_2_MO_6_ and Bi_2_Mo_2_O_9_ has the greatest absorption range (Figure 6). The band gap energy (E_g_) of Bi_2_WO_6,_ Bi_2_W_2_O_9_, Bi_2_MoO_6_ and Bi_2_Mo_2_O_9_ were computed to be 2.78, 2.76, 2.72, and 2.70 eV respectively and exhibit in the inset of Figure 6. The result indicated that Bi_2_M_2_O_9_ samples presented an enhanced absorbance ability compared to Bi_2_MO_6_.

### 2.5. Photocatalytic Properties

**Figure 7:**
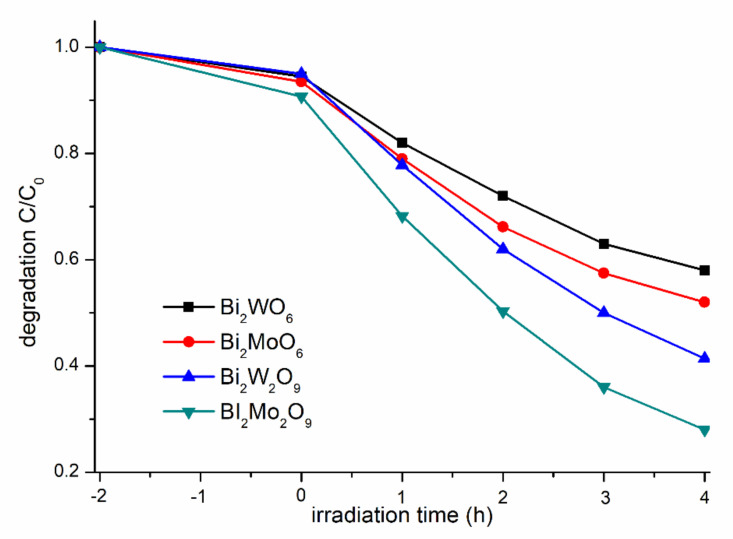
Comparison of absorption and degradation rate of MB using Bi_2_WO_6,_ Bi_2_W_2_O_9_, Bi_2_MoO_6_ and Bi_2_Mo_2_O_9_.

The photocatalytic performance of the as-synthesized photocatalysts were evaluated by examining the photodegradation of MB solution under visible-light irradiation (Figure 7). Generally, the different concentrations of MB adsorbed on the catalyst surface will have a great influence on the photocatalytic performance, so the adsorption ratio was collected when adsorption–desorption equilibrium was achieved before irradiation [35]. Bi_2_WO_6,_ Bi_2_W_2_O_9_, Bi_2_MoO_6_, and Bi_2_Mo_2_O_9_ samples presented a similar capacity for MB absorption, and it can be obviously observed that Bi_2_M_2_O_9_ samples exhibit a higher photocatalytic activity than that of Bi_2_MO_6_. Bi_2_Mo_2_O_9_ displayed the best photocatalytic activity among the four test samples, and the photodegradation rate reached up to 75% within 4 h.

**Figure 8:**
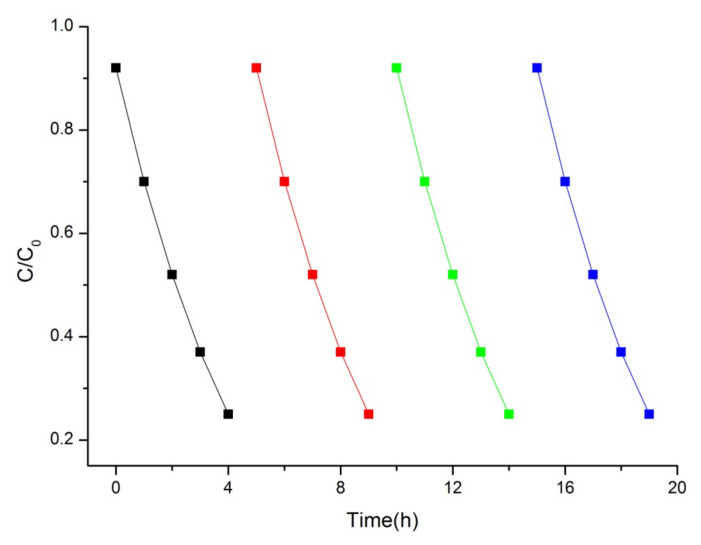
Cyclic photocatalytic activity of MB by Bi_2_Mo_2_O_9_ photocatalyst.

In our work, the cycling experiment for MB photocatalytic degradation was performed under visible-light irradiation to evaluate the stability of the best photocatalyst (Bi_2_Mo_2_O_9_). As shown in Figure 8, the photocatalytic performance of Bi_2_Mo_2_O_9_ did not display any significant reduction for MB degradation. This result confirms that Bi_2_M_2_O_9_ photocatalysts are not easily photo-corroded during the photodegradation of the pollutant molecules, which is important for their application.

### 2.6. Photocatalytic Mechanism

**Figure 9:**
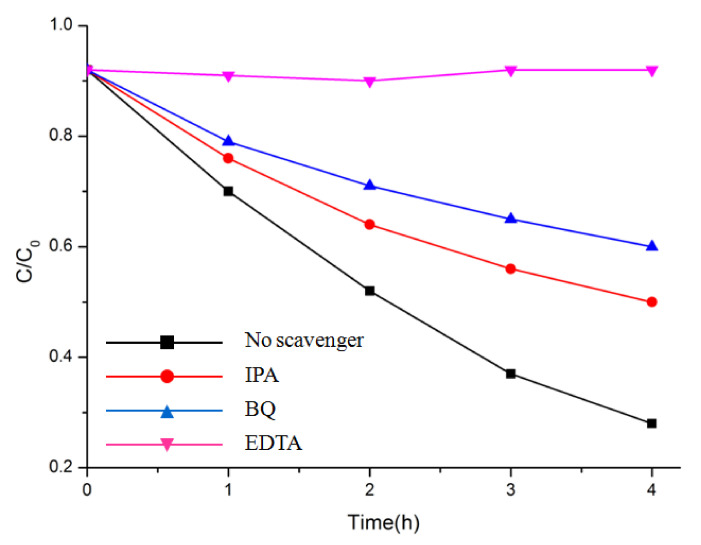
Photocatalytic degradation of MB over Bi_2_Mo_2_O_9_ photocatalyst with the addition of scavengers EDTA, BQ, and IPA.

Overall, the photocatalytic activity of Bi_2_M_2_O_9_ was highly improved compared to ordinary Bi_2_MO_6_. As noted above, such enhancement may partially come from structure modification. To understand the structure modification effect on Bi_2_M_2_O_9_ and the charge behavior during the photocatalyst process, trapping experiments with different scavengers were performed with Bi_2_Mo_2_O_9_ (Figure 9). In this way, active species could be determined, including holes (h^+^), superoxide radicals (˙O_2_^−^) and hydroxyl radicals (˙OH) with effective oxidation and reduction potentials [36,37,38]. In the present study, isopropyl alcohol (IPA), ethylenediaminetetraacetic acid disodium salts (EDTA), and 1,4-benzoquinone (BQ) were used as scavengers of ˙OH, h^+^, and ˙O_2_^−^, respectively. Remarkably, MB degradation was halted as we added the scavenger EDTA (1 mM) for h^+^ to the reaction system. Meanwhile, the photodegradation rate of MB evidently declined with the addition of the scavenger BQ (1 mM) for ˙O_2_^−^. However, there was no clear reduction in the degradation rate of MB when the scavenger IPA (1 mM) for ˙OH was added. These results indicate that h^+^ and ˙O_2_^−^ were the main reactive species, while ˙OH had little influence on the MB degradation process.

**Figure 10:**
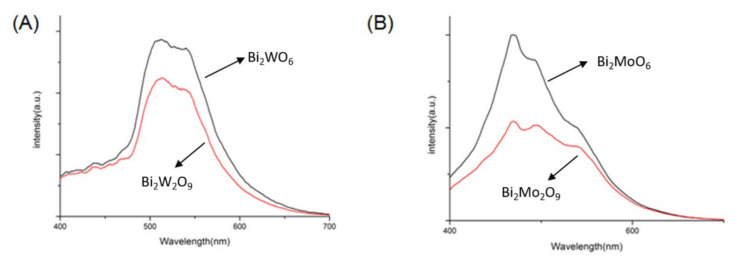
Photoluminescence (PL) spectra of (**A**) Bi_2_WO_6_ and Bi_2_W_2_O_9_; (**B**) Bi_2_MoO_6_ and Bi_2_Mo_2_O_9_.

The electron–hole separation condition was tested by PL in the range of 400–700 nm. As shown in Figure 10, compared to Bi_2_MO_6_, the PL intensities of Bi_2_M_2_O_9_ were perceptibly weaker, and Bi_2_Mo_2_O_9_ has the lowest peak intensity. Usually, a lower PL intensity shows stronger photogenerated charge separation, which leads to excellent photocatalytic efficiency of the photocatalyst [39]. The experiment results reveal that Bi_2_M_2_O_9_ has an elevated ability of electron–hole separation.

**Figure 11:**
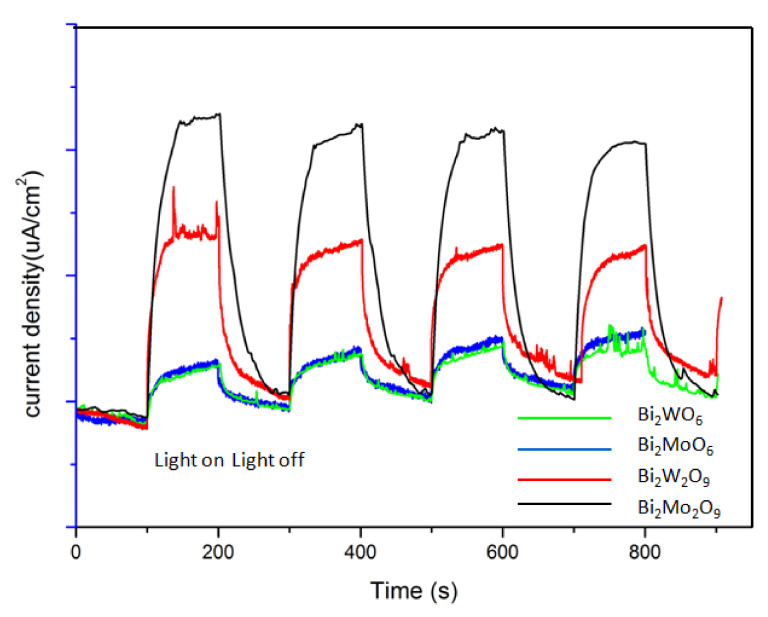
Photocurrent responses of Bi_2_WO_6,_ Bi_2_W_2_O_9_, Bi_2_MoO_6_ and Bi_2_Mo_2_O_9_ samples under visible-light irradiation.

The photocurrent responses directly related to the generation and transfer of the photogenerated electrons and holes [40,41]. Figure 11 shows the photocurrent response of Bi_2_WO_6,_ Bi_2_W_2_O_9_, Bi_2_MoO_6_ and Bi_2_Mo_2_O_9_ samples. Obviously, the current abruptly increased and decreased through on–off cycles under visible-light irradiation. Bi_2_M_2_O_9_ samples showed a significantly improved photocurrent response compared to that of the Bi_2_MO_6_ samples. This suggests that more efficient separation of the photogenerated charge carriers occurred in Bi_2_M_2_O_9_ samples. In addition, it should be noted that Bi_2_M_2_O_9_ samples hold obvious residual currents when the light source is switched off. This is probably attributed to the modified structure; it may lead to some remnant electron–hole pairs when the visible-light source is removed, which could release the trapped electrons or holes because of the self-thermal motion.

Based on the characterization methods above, a possible photocatalytic mechanism of the Bi_2_M_2_O_9_ photocatalyst under visible-light irradiation is therefore proposed, and the Bi_2_M_2_O_9_ photocatalyst has a stable double Rigid Unit (RU), which consists of two octahedral layers. Double RU mode can be regarded as a strong electron-drawing group, and Bi_2_M_2_O_9_ show a stable reduced bond distance of octahedral RU. The influence of modified structure is reflected in PL test and photocurrent measurement. The reduction of PL intensity and the enhancement of photocurrent express promoted photogenerated electron–hole pairs separation ability; the strong electron-drawing group traps the photogenerated electrons and leads to the increase of active species to promote the photocatalytic performance.

**Figure 12:**
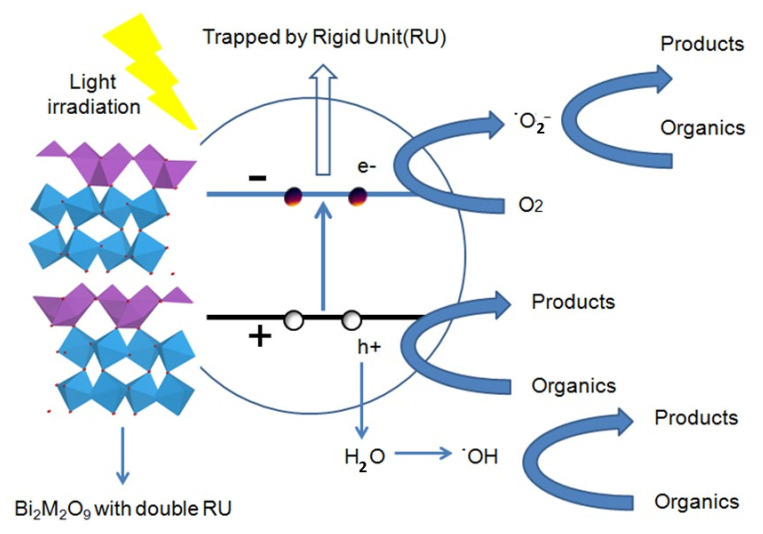
Schematic illustration of the mechanism of Bi_2_M_2_O_9_ photocatalyst under visible-light irradiation.

As shown in Figure 12, the photogenerated electrons could migrate to the conduction band (CB) from the valence band (VB) and the photogenerated holes formed in the valence band when the semiconductor was irradiated with visible light. The shortened bond distance of octahedral RU generates a strong electron-drawing efficiency that traps the electron firmly and reduces the recombination rate. Evidentially, the trapped electrons could easily transfer to the oxygen molecules (O_2_) adsorbed on the surface of the Bi_2_M_2_O_9_ catalysts. Subsequently, the released electrons react with O_2_ to form the active superoxide radical anion species (O_2_^−^). The electron capture and release process enhances the charge transfer and separation efficiency of photogenerated electrons and holes, which contributes to organic contaminant photodegradation by the h^+^ and ˙O_2_^−^ species.

Therefore, the above process indicates that an appropriate increase the number of (Bi_2_O_2_)^2^^+^ layers and (W_2_O_7_)^2^^−^ layers to form a stable Rigid Unit (RU) mode could significantly improve photocatalytic activity.

## 3. Experimental

### 3.1. Synthesis of the Photocatalysts

All the reagents used in this study were of analytical purity (Sinopharm Chemical Reagents Co., Ltd., Shanghai, China) and used without any further purification. Bi_2_WO_6_, Bi_2_W_2_O_9_, Bi_2_MoO_6_ and Bi_2_Mo_2_O_9_ photocatalysts were prepared through a hydrothermal method and the general synthesis processes were as follows: proportionate amounts of sodium tungstate (Na_2_WO_4_·H_2_O) or sodium molybdate (Na_2_MoO_4_·H_2_O) were dissolved in Milli-Q water. Then proportional bismuth (III) nitrate pentahydrate (Bi(NO_3_)_3_·5H_2_O) was dissolved in nitric acid and anionic surfactant AOT was introduced into the solution sequence under stirring to form a transparent solution and ensure Bi:Mo and Bi:W keeps 2:1 during the preparation process of Bi_2_WO_6_ and Bi_2_MoO_6_, similarly, a value of the ratio of Bi and W or Mo stays at 1 when preparing Bi_2_W_2_O_9_ and Bi_2_Mo_2_O_9_. We adjust the pH value of the solution to ca. 7 using NaOH solution. The slurry was stirred for another 30 min and then poured into Teflon-lined stainless steel autoclaves. The sealed reactors were then heated at 180 °C for 12 h. Then, the products were cooled to room temperature naturally and collected via centrifugation and washed in Milli-Q water and ethanol several times to make sure that the residual impurities were all removed, and then the product was dried at 80 °C for 8 h.

### 3.2. Characterization of the Photocatalysts

The crystalline phases of the as-prepared samples were analyzed using X-ray diffraction (XRD) (D/MAX-RB, Rigaku, Tokyo, Japan). The diffraction patterns were recorded in the 2θ range from 10 to 70° with a Cu Kα source (λ = 1.5418 Å) running at 40 KV and 30 mA. The morphology images of the as-prepared samples were captured using scanning electron microscopy (SEM) equipped with an energy-dispersive X-ray spectrometer (EDX) on a SUPRA 55 SAP-PHIRE instrument operating at 20 KV. High-resolution transmission electron microscopy (HRTEM) images were acquired with a transmission electron microscopy (F-20, FEI, Hillsboro, OR, USA) at an accelerating voltage of 200 KV. The UV-Vis diffuse reflectance spectra of the as-prepared samples were examined at room temperature using a UV-Vis spectrophotometer (T9s; Persee, Beijing, China) equipped with an integrating sphere. BaSO_4_ was used as the blank reference. Photoluminescence (PL) spectra were recorded using a fluorescence spectrophotometer (F-4500; Hitachi, Tokyo, Japan) with a Xe lamp as the excitation light source. X-ray photoelectron spectroscopy (XPS) was examined on an X-ray photoelectron spectrometer (ESCALAB 250Xi, Thermo Scientific, Waltham, MA, USA) using an Al Kα radiation.

### 3.3. Photocatalytic Experiment

The photocatalytic activities of Bi_2_WO_6,_ Bi_2_W_2_O_9_, Bi_2_MoO_6_, and Bi_2_Mo_2_O_9_ photocatalysts under visible light were assessed by degrading 10 mmol·L^−1^ methylene blue (MB). A 400 W Xe lamp with a UV-cut-off filter (λ > 420 nm) was used as a light source and set about 10 cm apart from the reactor. The experiments were as follows: 40 mg of the photocatalyst was dispersed in 40 mL of MB solution. It was then stirred for 120 min in the dark to achieve an adsorption–desorption equilibrium before light irradiation. During the irradiation, the reaction samples were collected at 60 min intervals and centrifuged to separate out the photocatalyst particles. The ratios (C/C_0_) of the MB were adopted to evaluate the degradation efficiency (i.e., C_0_ was the initial concentration, where C was the concentration at a certain time) by checking the absorbance spectrum at 664 nm for MB using a UV-Vis spectrophotometer (T9s; Persee, Beijing, China).

### 3.4. Measurement of Photocurrent

The measurement of the photocurrent was carried out with an electrochemical workstation (5060F, RST, Zhengzhou, China) in a standard three-electrode system including the samples, an Ag/AgCl electrode (saturated KCl), and a Pt filament used as the working electrode, reference electrode, and counter electrode, respectively. In addition, a pre-made 0.5 mol L^−1^ Na_2_SO_4_ aqueous solution was introduced as the electrolyte. A 100 W incandescent lamp with a 420 nm cut-off filter was used as the light source. The working electrode was manufactured as follows: 5 mg samples were appended to 2 mL of ethanol and Nafion mixture solution (*v*/*v* = 30:1), followed by spreading on the middle of an ITO glass in a rounded hole with a diameter of 6 mm.

## 4. Conclusions

In this study, ordinary Bi_2_WO_6_ and Bi_2_MoO_6_ samples were successfully synthesized; furthermore, a novel Bi_2_Mo_2_O_9_ fastener sphere and a Bi_2_W_2_O_9_ irregular polyhedron were prepared by a hydrothermal method with AOT introduced. The results revealed that structure-modified Bi_2_W_2_O_9_ and Bi_2_Mo_2_O_9_ exhibited enhanced photocatalytic performance. The Rigid Unit (RU) and modified bond structure influence the trapping–release process of electrons to promote the separation efficiency of photogenerated electron–hole pairs. Therefore, an appropriate increase of the layer number of (W_2_O_7_)^2^^−^ or (Mo_2_O_7_)^2^^−^ between (Bi_2_O_2_)^2^^+^ layers to form an RU strong electron-drawing group as a method of structure modification can be a potential strategy to improve visible-light photocatalytic activity by affecting the charge behavior.

## Figures and Tables

**Table 1:** EDX data of Bi_2_WO_6_, Bi_2_W_2_O_9_, Bi_2_MoO_6_ and Bi_2_Mo_2_O_9_.

Sample	Element	Wt%	At%
Bi_2_WO_6_	Bi	58.82	22.28
	W	27.90	12.02
	O	13.28	65.70
Bi_2_W_2_O_9_	Bi	24.48	11.73
	W	24.32	13.25
	O	11.98	75.02
Bi_2_MoO_6_	Bi	67.66	21.56
	Mo	16.20	11.25
	O	16.14	67.19
Bi_2_Mo_2_O_9_	Bi	54.13	14.62
	Mo	26.01	15.30
	O	19.86	70.07

## Data Availability

The data presented in the study are available from the corresponding author.

## Sample Availability

Samples of the compounds are not available from the authors.
